# Learning a spatial-temporal texture transformer network for video inpainting

**DOI:** 10.3389/fnbot.2022.1002453

**Published:** 2022-10-13

**Authors:** Pengsen Ma, Tao Xue

**Affiliations:** School of Computer Science, Xi'an Polytechnic University, Xi'an, China

**Keywords:** transformer, video inpainting, texture converter, spatial-temporal, deep learning

## Abstract

We study video inpainting, which aims to recover realistic textures from damaged frames. Recent progress has been made by taking other frames as references so that relevant textures can be transferred to damaged frames. However, existing video inpainting approaches neglect the ability of the model to extract information and reconstruct the content, resulting in the inability to reconstruct the textures that should be transferred accurately. In this paper, we propose a novel and effective spatial-temporal texture transformer network (STTTN) for video inpainting. STTTN consists of six closely related modules optimized for video inpainting tasks: feature similarity measure for more accurate frame pre-repair, an encoder with strong information extraction ability, embedding module for finding a correlation, coarse low-frequency feature transfer, refinement high-frequency feature transfer, and decoder with accurate content reconstruction ability. Such a design encourages joint feature learning across the input and reference frames. To demonstrate the advancedness and effectiveness of the proposed model, we conduct comprehensive ablation learning and qualitative and quantitative experiments on multiple datasets by using standard stationary masks and more realistic moving object masks. The excellent experimental results demonstrate the authenticity and reliability of the STTTN.

## 1. Introduction

Video inpainting involves smearing moving or stationary objects in a video frame sequence using masks. The smeared parts are filled back based on the current frame and the content information of other frames of the video, and the repaired video should have the effect that the smeared positions 'disappear'. Typical applications are video restoration (Kim et al., 2018; Chang et al., 2019a,b), watermark removal (Zou et al., 2021), object removal (Perazzi et al., 2016; Chang et al., 2019d), etc. The closer the smeared area is to the actual video after being repaired, the better the repair effect.

Video inpainting needs to combine time domain and spatial domain information to process video frames. The spatial information in the current frame is searched, followed by the appropriate frames in other frames as reference frames to search the time domain information. Finally, the two parts of information are integrated and filled back into the original frame to complete the repair of the mask position (Zeng et al., 2020; Liu et al., 2021a). Video inpainting should first consider whether the missing information of the current frame is 'exposed' in other frames. If the missing information of the current frame is found in other frames, then the current frame should be used as a reference frame. Valuable features should be matched, extracted, and transmitted to the input frame as information to repair the mask position. Although the recent appearance of deep learning has made significant progress in the image and video inpainting (Iizuka et al., 2017; Boßmann et al., 2019; Yu et al., 2019), a model's ability to capture useful information and reconstruct it in video frames is still fragile (Chang et al., 2019a; Lee et al., 2019; Oh et al., 2019; Xu et al., 2019).

In summary, video inpainting needs to integrate the information acquired in time and space and effectively transform and fill it back into the restored image. The more complicated and challenging portion of this process is 2-fold: What information (in time) should be extracted from the reference frame? How can the information of the reference frame and the current frame be effectively extracted and used (spatially)?

We put forward the spatial-temporal texture transformer network to solve these problems in video inpainting. STTTN is divided into the following six parts: (1) Make the feature similarity measure more accurate and frame pre-repair. (2) By introducing Regional Normalization (RN) (Yu et al., 2020), spatial pixels are divided into different regions according to a mask, which solves the problem of the deviation of the mean and variance, thus constructing an encode with stronger information extraction ability. (3) To embed the information in the image, similar to the standard transformer structure (Dosovitskiy et al., 2020; Liu et al., 2021c), a related texture information embedding module (RE) is introduced to embed the reference and input frames. (4) Coarse low-frequency feature transfer (CLFT) is used to convert low-frequency information such as contours from reference frames to input frames. (5) Refinement high-frequency feature transfer (RHFT) is used to transfer further delicate texture information such as image details to the input frame and perfect the repair mask. (6) Information such as the feature texture, which is composed of various pieces of information, is obtained. Similar to the encoder, we added learnable region normalization in the decoder to help the fusion of corrupted and uncorrupted areas and more stable modified video frames.

These six parts help each other build a powerful space-time transformer video restoration network. There are three steps in the inpainting: the input frame and reference frames before the encoder are pre-repaired, the coarse low-frequency texture features are transferred and repaired, and finally the high-frequency texture features related to details are further refined and repaired. The three parts are paved from low to high, and the complete repair process is formed layer by layer.

To verify the progressive nature of the STTTN, we carried out a large number of qualitative and quantitative experiments. An ablation study of four parts of the texture converter and loss function proved the effectiveness of each part of the components.

Our research makes the following contributions:

- A novel and effective spatio-temporal texture converter for video restoration, which achieves significant improvements over the state-of-the-art approaches, is proposed.
- Regional normalization (RN) introduced into the encoder and decoder creatively stabilizes the effect of video restoration.
- From the results of various mask experiments on multiple datasets, STTTN achieves excellent results visually and in terms of evaluation parameters.

## 2. Related studies

Video Inpainting refers to smearing some fixed areas or moving objects in a video and filling the smeared areas back in a generated way. It is required as far as possible to leave no trace (that is, it is not easy to detect by the naked eye). Image inpainting fills the missing area in a single image. In a narrow sense, image inpainting is a subset of video inpainting. The difference between them is that video inpainting needs to integrate the temporal and spatial information acquired from all video frames and effectively transform and fill it back into the restored image. Image inpainting only needs to make use of the spatial information outside the mask of the current picture without considering the information of other frames because it is only a single picture without any temporal information. From the perspective of repair methods, video inpainting is mainly divided into two methods. One is explicit repair, that is, it acts directly on the image and repairs from the pixel level. The other is implicit inpainting, that is, it acts on image coding and inpainting from the feature level.

### 2.1. Explicit inpainting

Explicit inpainting is a network design pattern of 'graph to graph' and is directly constructed. Before the popularity of deep learning, the main image and video inpainting methods were diffusion-based and patch-based methods. The central idea of diffusion-based methods (Ballester et al., 2001; Levin et al., 2003) was to predict and fill holes according to the pixels around the region to be filled. For example, the fast marching method (FFM) (Telea, 2004), and the fluid dynamics method (FDM), are classic diffusion-based inpainting algorithms, but their limitations are also pronounced. It is more suitable for image pinhole inpainting with little color change and a simple scene. Patch-based methods complete the repair task by finding the most appropriate area to fill the hole and pasting it into the place to be inpainting. For a single image, it searches for the most suitable area outside the hole area, that is, spatial-based inpainting. Video inpainting considers all regions in the current frame and other frames to select the most appropriate area to fill, which is a temporal-based and spatial-based approach. Generally speaking, both diffusion-based and patch-based methods are predicted or pasted to the area to be inpainting according to the area around the hole. They can not capture advanced semantic information. They are repaired in a way that can't be learned, so they are also called Non-learning-based inpainting.

The appearance of deep learning makes up for the deficiency of content restoration in complex and dynamic motion areas from multiple objects. At present, the primary display inpainting methods based on deep learning are divided into the following two types. The first is optical flow calculation, that is, the 'movement trend' of pixels is calculated based on the difference between the front frame and the back frame. This 'trend' is used to predict the color propagation to fill the missing mask block (Xu et al., 2019; Lao et al., 2021). The second is 3D-CNN/RNN, which directly stacks the frames in time series according to the number of channels to form a large matrix for convolution calculation (Kim et al., 2019; Wang et al., 2019). This method is cumbersome, takes up considerable video memory, and consumes many computing resources. In addition, the effect worsens when encountering some frequently switched and complex video scenes, so there is little room for improvement.

### 2.2. Implicit inpainting

Traditional restoration methods often consume too much memory and lead to a long reasoning time, and can not effectively capture texture information in the temporal domain and spatial domain. At the same time, a network based on implicit inpainting is small and exquisite, with a relatively strong effect and ample room for improvement. The depth representation of the image is obtained using an encoder. Then, a series of patching operations are performed on the image representation: attention (Tang et al., 2019; Liu et al., 2021a; Shu et al., 2021; Zou et al., 2021), generative adversarial network (Chang et al., 2019a,d; Zou et al., 2021), gated convolution (Yu et al., 2019), region normalization (Suin et al., 2021), etc. This is mapped (decoder) back to the image to generate video frames after patching is completed.

Video inpainting needs to find high-level semantic information in time and space; that is, it needs to capture long-distance dependencies. In recent years, the appearance of the transformer (Arnab et al., 2021; Chen et al., 2021; Wang and Wang, 2022) has provided a new solution for vision tasks. Compared with traditional CNN (Gu et al., 2022) and RNN-based (Lin et al., 2022) methods, transformers have better capability to understand shape and geometry and capture the dependencies between long distances. We propose a spatial-temporal texture transformer network (Han et al., 2020). We learn the feature relationship between video frames and within frames according to the semantic consistency of context to complete hole filling, which is effective and efficient for video inpainting.

## 3. Approach

First, we introduce the overall architecture design of STTTN and then explain its five essential components in detail, namely encoder, relevance embedding (RE), coarse low-frequency feature transfer (CLFT), refining high-frequency feature transfer (RHFT), and decoder. Finally, we combed the whole video restoration process and explained the model's loss function more clearly through pseudo-code.

### 3.1. Overall design

We use implicit inpainting to repair the video from the feature level. To determine the defects of the previous architecture and achieve a better inpainting effect, we erase the time domain search part of the previous baseline model (Zeng et al., 2020; Liu et al., 2021a,b) and find that the inpainting ability is significantly reduced and even lags behind the effect of many nonspatiotemporal video inpainting models. This shows that most of the previous study explored how to search the memory in the temporal domain but neglected to examine the depth representation of the obtained images; that is, only by introducing temporal domain search can the model perform better, but their spatial domain search is not as good as the original image inpainting model, which needs stronger information extraction ability and fine content reconstruction ability. Therefore, we built a new encoder and decoder architecture, which makes STTTN have a more vital ability to capture image structure and information in the time domain. The overall architecture idea is that the depth representation of the image is obtained by the encoder. Then, after the image representation is repaired, the image is mapped back to the decoder to generate the repaired image frame.

**Figure 1:**
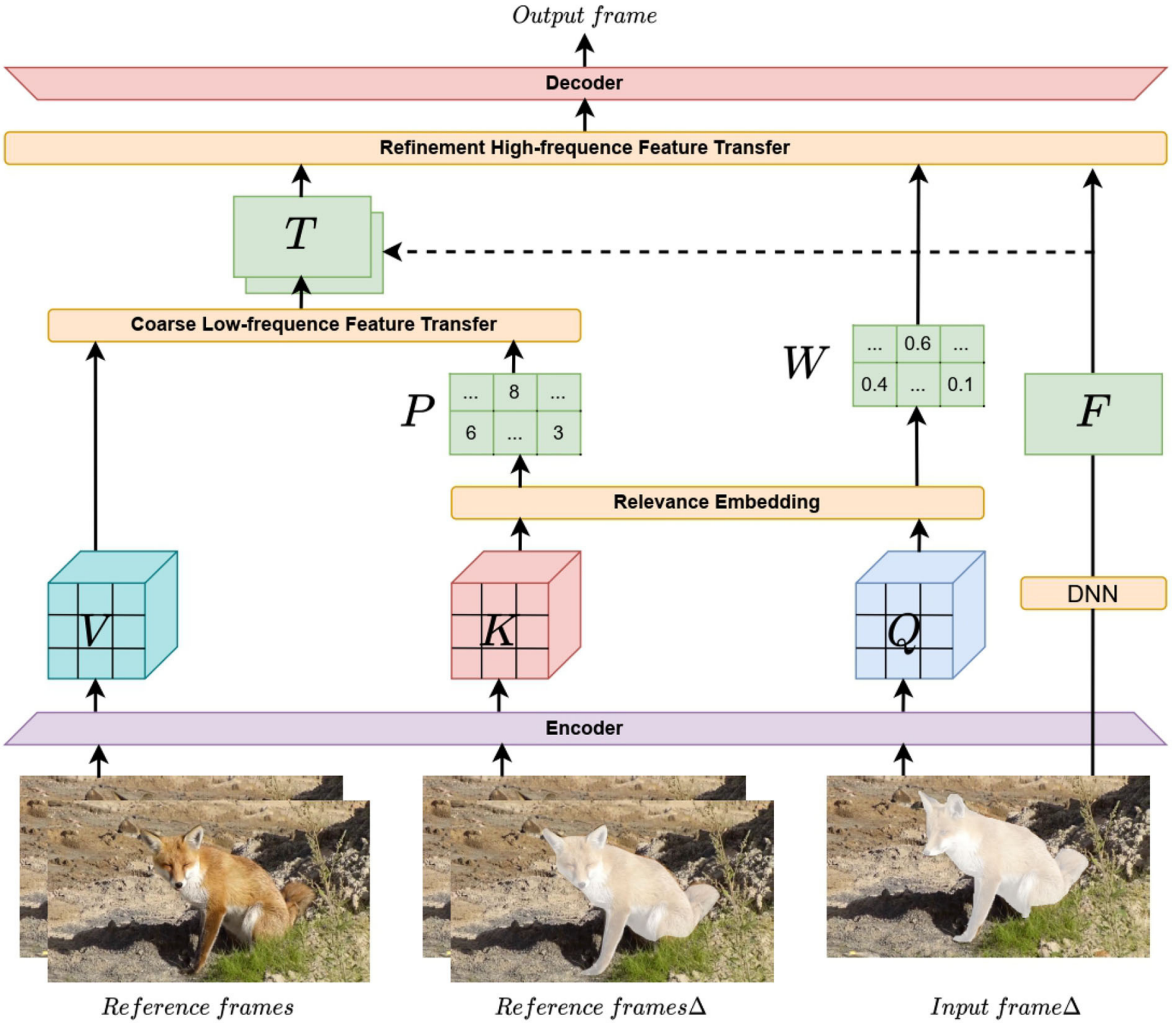
Overview of spatial-temporal texture transformer network (STTTN) structure.

The overall structure of the spatial-temporal texture transformer is shown in Figure 1. This structure contains six parts. The first part is the preprocessing of video frames before they are input into the network. The input framesΔ and reference framesΔ represent the pre-repaired input frames and reference frames, respectively. Precisely, we mask at the position where the reference frames are consistent with input frames and prerepair the mask to obtain reference framesΔ to ensure domain consistency with input framesΔ. Here, we fix the random seed to ensure that the mask position and size of the input frame and the reference frame are consistent, and then we randomly switch the random seed to generate a new mask when processing the next frame. It is more accurate to measure the feature similarity with the domain (Yang et al., 2020), in which the prerepair method is a fast marching method (FFM) (Telea, 2004). The remaining five parts are an encoder with super information extraction ability, embedding module (RE) to find a correlation, coarse low-frequency feature transfer (CLFT), refinement of high-frequency feature transfer (RHFT), and accurate content decoder with reconstruction capability. Details are discussed below.

### 3.2. Spatial-temporal texture transformer

#### 3.2.1. Encoder

**Figure 2:**
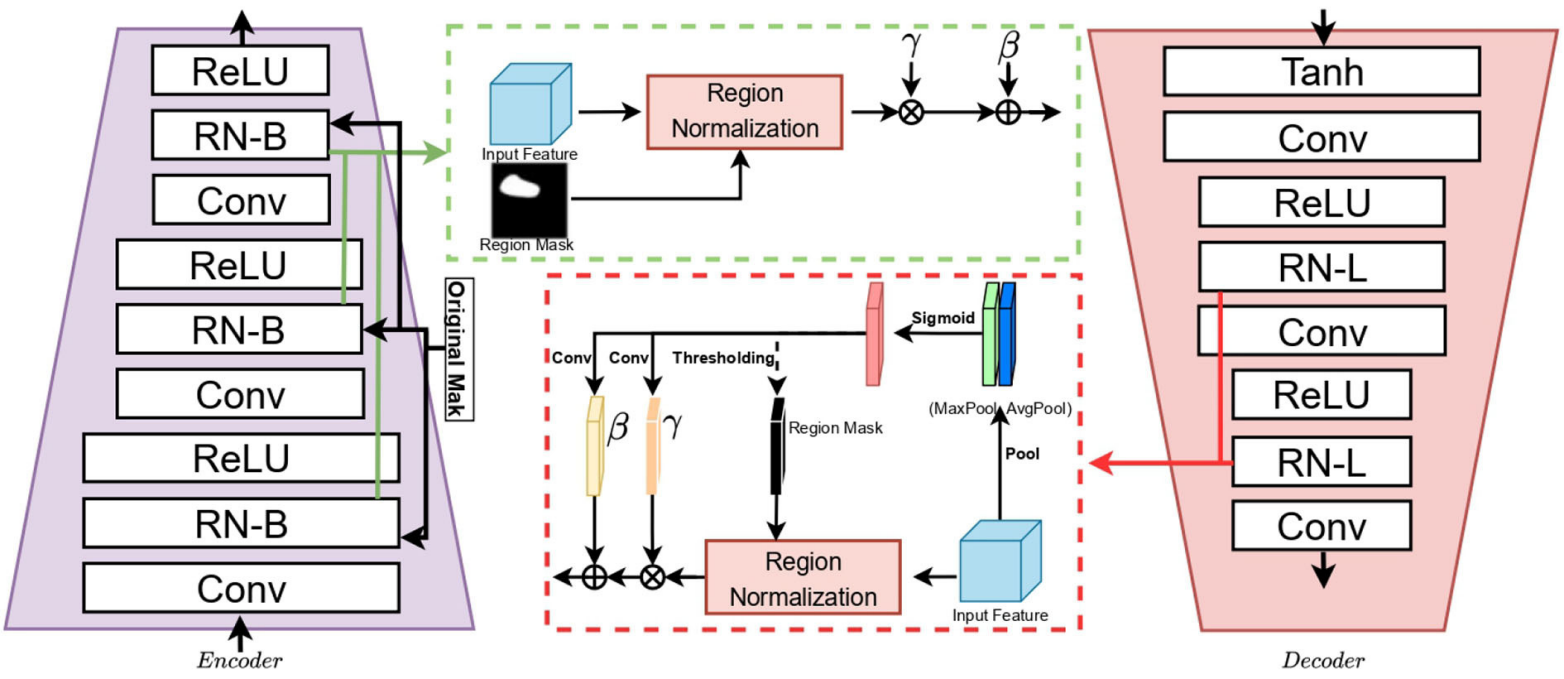
Structure of encoder and decoder.

The traditional video processing and image inpainting methods use feature normalization (FN) to help with network training (Kobla et al., 1996; Wang et al., 2020), but they are often performed on the entire frame without considering the impact of pixels in the corrupted region on the mean/variance. By introducing regional normalization (RN), the spatial pixels are divided into different regions according to the mask, and then the mean and variance are calculated in different regions. As shown in Figure 2, we embed basic regional normalization (RN-B) on the encoder, which normalizes the corrupted and uncorrupted regions based on the input mask. This allows mean and variance offsets to be more accurate, which is more conducive to obtaining a deep image representation, which can more comprehensively extract helpful information from video frames.

The input to the encoder network consists of three frames (input framesΔ, reference framesΔ, and reference frames). Inp, InpΔ, Ref, and RefΔ represent the input frames, input framesΔ, reference frames, and reference framesΔ, respectively. InpΔ and RefΔ consist of an RGB image, hole mask, and no-hole mask. The hole mask on the RGB image is a single-channel greyscale image, and the no-hole mask is an area other than the hole mask area. These inputs are concatenated along the channel axis to form a 5-channel image before being fed into the first layer. Ref are composed of three-channel RGB images. Given an input feature *F*∈*R*_*C*×*H*×*W*_ and binary region mask *M*∈*R*_1 × *H*×*W*_ that indicates a corrupted region, for each channel, there are two sets of learnable parameters γ and β for the affine transformation of each region. *Via* the encoder, the obtained image representation consists of three parts: *Q* (representative attention information), *K* (attention information of memory frame), and *V* (representative content information of memory frame).

#### 3.2.2. Relevance embedding

Different from the previous operation of obtaining q, k, v through a linear transformer and then calculating attention (Lee et al., 2021), we obtain Q, K, V with sufficient texture feature information through the encoder, which makes it easier to find the correlation between the input frame and the reference frames in the time domain. First, we use RE to estimate the similarity between Q and K, so as to establish the correlation between Inf and Ref.We unfold Q and K into patches, denoted as *q*_*i*_(*i*∈[1, *H*_*Inp*_×*W*_*Inp*_]) and *k*_*j*_(*j*∈[1, *H*_*Ref*_×*W*_*Ref*_]). We calculate their similarity by dot multiplying *q* and *k*^*T*^, where *T* represents the transpose operation:


(1) $$\begin{array}{l} R_{i,j}=q_{i}\times k_{j}^{T} \end{array}$$

The larger *R*_*i, j*_, the stronger the correlation between the two feature blocks, and the more texture information can be migrated, and vice versa.

With the correlation *R*_*i, j*_ obtained by RE, we can obtain two parts *P* and *W* for coarse low-frequency feature transfer and refinement of high-frequency feature transfer, respectively. The specific calculation details are in Sections 3.2.3, 3.2.4.

#### 3.2.3. Coarse low-frequency feature transfer

To transfer the low-frequency information of images, such as contours from the reference frame to the input frame, we designed the coarse low-frequency feature transfer (CLFT). The previous attention mechanism converted *R*_*i, j*_ through softmax into a weight directly and then multiplied the weight by *V*, which is a weighted average of *V*:


(2) $$\begin{array}{l} \mathrm{Attention}\left(Q,K,V\right)=\mathrm{softmax}\left(\frac{QK^{T}}{\sqrt{d_{k}}}\right)V \end{array}$$

However, doing so may transfer a large number of textures that are not useful for the input frame to the target frame, resulting in blurring of the repaired area. To improve the ability to transfer low-frequency texture features for all reference frames. we will correlate the coarse low-frequency features in *V* over different temporal domains with input frames *via* CLET. More specifically, we first calculate a coarse low-frequency feature transfer map *P* in which the *i*-th element *P*_*i*_(*i*∈[1, *H*×*W*]) is calculated from the relevance *R*_*i, j*_:


(3) $$\begin{array}{l} P_{i}=\arg\max r_{i,j} \end{array}$$

That is, each value *p*_*i*_ in map *P* represents the most relevant position index of a frame on all reference frames with the *i*-th position of the input frame. The specific calculation process obtains the index corresponding to the maximum value through the second item of the return value of the torch.max() function. After obtaining the most relevant position index, we extract the low-frequency texture features that should be transferred most, so we only need to take the position of the frame that needs to be transferred in the patch *v* of the unfolded, then we can get the texture feature map *T*, where each position of *T* contains the high-frequency texture features of the most similar position in the Ref, where *t*_*i*_ represents the value of the *i*-th position of *T*:


(4) $$\begin{array}{l} t_{i}=v_{p_{i}} \end{array}$$

We obtain a rough feature representation T for input frames, which is then used in our refinement of high-frequency feature transfer (RHFT).

#### 3.2.4. Refinement high-frequency feature transfer

High-frequency detail information is also essential for video inpainting (Bishop et al., 2003), so we designed a refinement of high-frequency feature transfer (RHFT). To fuse the most suitable high-frequency texture in the temporal and spatial domains with the input frame, a weight matrix *W* is calculated from *R*_*i, j*_ to represent the confidence of the transferred texture features for each position in *T*. The specific calculation process for obtaining *W* is to obtain the maximum value of *R*_*i, j*_ through the first item of the return value of the torch.max() function, where *W* records the specific correlation of the most relevant feature block.


(5) $$\begin{array}{l} W_{i}=\max R_{i,j} \end{array}$$

To make full use of the original image information of the input frame, we divide the features of each level into two steps. First, the low-frequency texture features *T* of multiple frames in the temporal domain are obtained by CLFT, and the feature of the input frame is fusion. The product is then multiplied by the weight matrix *W*. At this time, *W* is equivalent to a weighted average of the features, which can more accurately transfer the texture features of the reference frame. Only two feature transfers cannot modify the input frame well, so we extract the features of the input frame again (Only two feature transfers cannot modify the input frame well, so we extract the features of the input frame again (feature *F* extracted by the DNN, which is a deep neural network composed of convolution and residual connections of many layers with convolution kernel of 3*3, and stride and padding are 1) and fuse them with high- and low-frequency features. The above operations can be expressed as the following formula:


(6) $$\begin{array}{l} F_{\text{out}}=F+\mathrm{Conv}\left(\mathrm{Concat}\left(T,F\right)\right)⊙\text{W} \end{array}$$

Conv, Concat, and ⊙ represent the convolution (the convolution operation adopted here is consistent with the convolution operation adopted by the DNN above), the concatenation operation, and the dot product, respectively, and Fout is the feature of the spatiotemporal texture output of the input frame combined with reference frames.

#### 3.2.5. Decoder

In the deep network, each corrupted area and uncorrupted area are increasingly difficult to distinguish, and the corresponding mask is difficult to obtain (Yu et al., 2020). To enhance the reconstruction ability of the image, we insert the learnable RN (RN-L) into the decoder to automatically detect the mask and nonmask. Regions are individually normalized, and a global affine transformation is performed to enhance their fusion. Finally, the repaired video frame is obtained by outputting the newly repaired representation through the decoder.

In summary, the STTTN can effectively transfer relevant high- and low-frequency texture features from the reference frames into the input frame, producing a more accurate mask filling process. For a more precise illustration of how we perform video inpainting, as in Algorithm 1, we detail the pseudocode of our entire calculation process:

**Algorithm 1:**
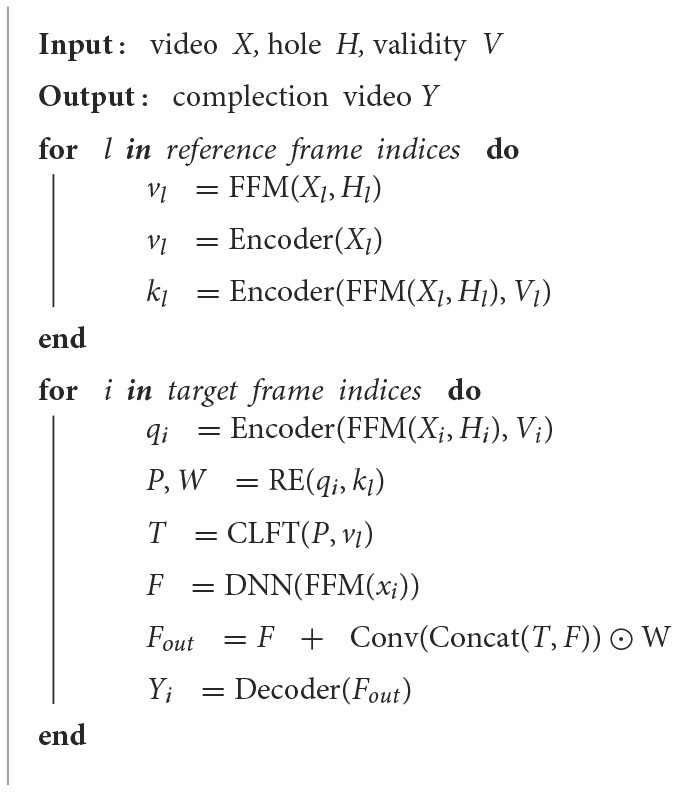
Spatial-temporal texture transformer network for video inpainting.

### 3.3. Loss function

The total loss consists of the following two components:


(7) $$\begin{array}{l} L_{\text{overall}}=L_{cha}+0.01L_{\text{per}} \end{array}$$

#### 3.3.1. Charbonnier loss

We abandon the L1 and L2 loss functions because they both cause the image to be too smooth and lack a sense of realism. Instead, we use a more stable loss function: the Charbonnier loss function (Lai et al., 2018a). It can be formulated as


(8) $$\begin{array}{l} L_{\text{cha}}=\sqrt{||I^{Out}-I^{Input}|{|}^{2}+\epsilon^{2}} \end{array}$$

where Input means original video, Out means synthesized video, and ϵ = 10^−3^ is a constant to avoid gradient disappearance and explosion.

#### 3.3.2. Perceptual loss

To make more effective use of texture features transferred from reference video, make the inpainted video frames more realistic, and maintain content invariance (Yang et al., 2020), we construct a perceptual loss that consists of two parts:


(9) $$\begin{array}{l} {ℒ}_{\text{per }}=\frac{1}{C_{i}H_{i}W_{i}}{\left\|\phi_{i}^{vgg}\left(I^{Out}\right)-\phi_{i}^{vgg}\left(I^{Ref}\right)\right\|}_{2}^{2}+\\ \;\frac{1}{C_{j}H_{j}W_{j}}{\left\|\phi_{j}^{Enc}\left(I^{Out}\right)-T\right\|}_{2}^{2} \end{array}$$

The first part is no different from ordinary perceptual loss (Johnson et al., 2016). $\phi_{i}^{vgg}$ represents the feature map of the *i*-th layer of VGG-16 pretrained on ImageNet (Deng et al., 2009); (*C*_*j*_, *H*_*j*_, *W*_*j*_) represents the number of channels, height, and width of the feature map of this layer; and *I*^*Ref*^ is the reference video for all frames. T is the texture feature transferred from V in Figure 2.

## 4. Experiments

### 4.1. Datasets and evaluation metrics

For a fair comparison of STTTN and other video inpainting models such as previous state-of-the-art versions, we use YouTube-VOS (Xu et al., 2018) and DAVIS (Caelles et al., 2018) as our datasets. The train/validation/test split is consistent with the original split. There are 3471, 474, and 508 video clips, respectively. For DAVIS, we divided its 150 video clips into 90 training sets and 60 validation sets and then randomly selected 30 as test sets.

To test the ability of the model to cope with a variety of practical application scenarios, we use two mask test models, namely, stationary masks and dynamic masks. The static mask means that the position of the fixed mask does not change, and the dynamic mask means that a moving object as a mask forces the mask keep the position transformation in each frame.

Various evaluation criteria are prerequisites to ensure the superior performance of the model. We use PSNR, SSIM, flow warping error (Lai et al., 2018b), video-based Fr'echet inception distance (VFID) (Wang et al., 2018), floating-point operations (FLOPs), and frames per second (FPS) as our evaluation metrics. VFID transfers the FID evaluation from the image to the video task, and the flow warping error measures the temporal stability of a video between the repaired frame and the original frame. FLOPs and FPS test the computing resources required by the model and the fluency of the repaired video, respectively.

### 4.2. Evaluation

To test the STTTN more comprehensively, we conduct qualitative and quantitative evaluations with five current SOTA methods: VINet (Kim et al., 2019), DFVI (Xu et al., 2019), LGTSM (Chang et al., 2019c), CAP (Lee et al., 2019), STTN (Zeng et al., 2020), and FGVC (Gao et al., 2020).

#### 4.2.1. Quantitative evaluation

As shown in Table 1, on the YouTube-VOS and DAVIS test sets, our proposed STTTN is generally at the highest level, and relatively few FLOPs and higher FPS ensure a lightweight model and the smoothness of the video.

**Table 1:** Quantitative results of video completion on YouTube-VOS and DAVIS datasets.

	**Accuracy**								**Efficiency**	

	**YouTube-VOS**				**DAVIS**				**FLOPs**↓	**FPS**↑
**Models**	**PSNR**↑	**SSIM**↑	**VFID**↓	**E**_*w*_ *arp↓*	**PSNR**↑	**SSIM**↑	**VFID**↓	**E**_*w*_ *arp↓*		
VINet	29.20	0.9434	0.072	0.1490	28.96	0.9411	0.199	0.1785	-	-
DFVI	29.16	0.9429	0.066	0.1509	28.81	0.9404	0.187	0.1880	-	-
LGTSM	29.74	0.9504	0.070	0.1859	28.57	0.9409	0.170	0.2566	261B	18.7
CAP	31.58	0.9607	0.071	0.1470	30.28	0.9521	0.182	0.1824	211B	15.0
STTN	32.34	0.9655	0.053	0.1451	30.67	0.9560	0.149	0.1779	233B	24.3
FGVC	31.28	0.9502	-	-	-	-	-	-	-	-
Proposed	32.68	0.9654	0.051	0.1421	31.32	211B0.9620	0.149	0.1738	254B	36.8

The bold red font represents the best score, and the blue font represents the second best score.

#### 4.2.2. Qualitative evaluation

**Figure 3:**
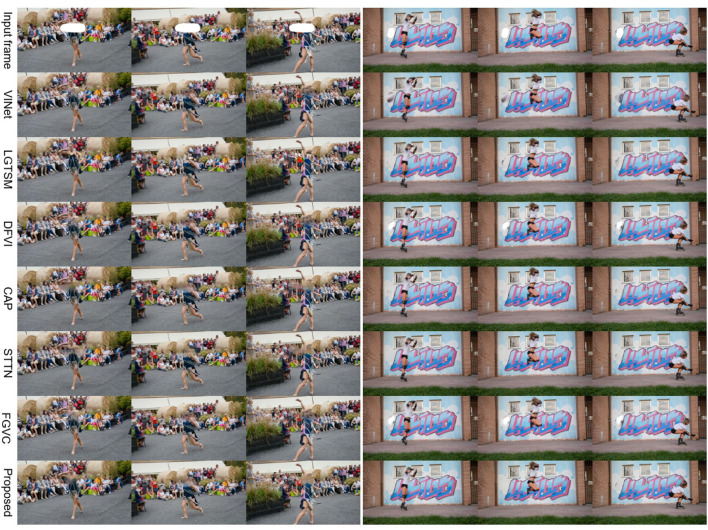
Qualitative comparison with other methods for stationary masks.

**Figure 4:**
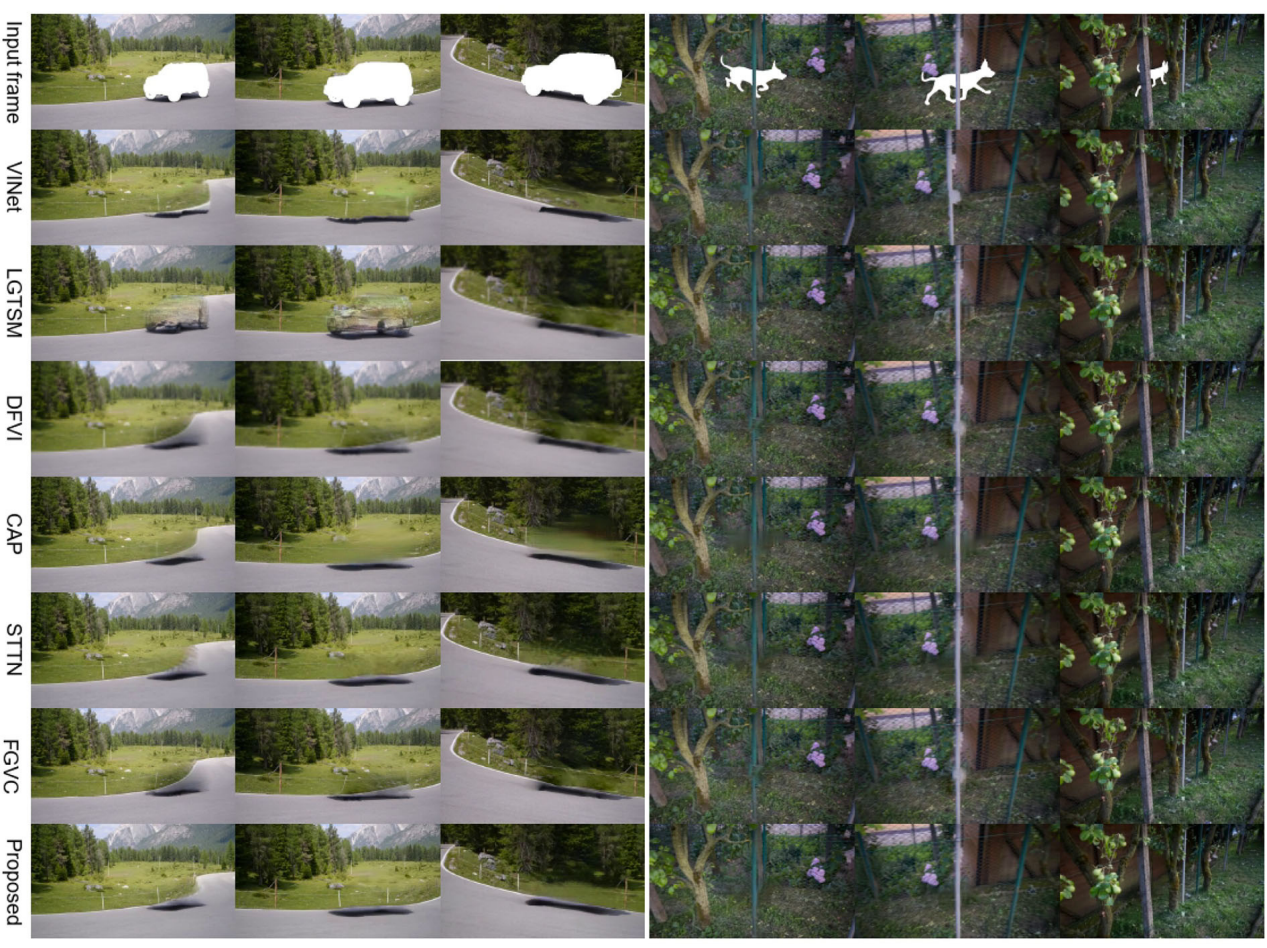
Qualitative comparison with other methods for dynamic masks.

To demonstrate the effectiveness and generalization of STTTN, as shown in Figures 3, 4, we conduct experiments on dynamic masks and static masks. It can be seen that compared with other current state-of-the-art models, regardless of whether it is a complex or straightforward scene, STTTN achieves the best restoration effect in terms of overall feeling and local details.

#### 4.2.3. User study

**Figure 5:**
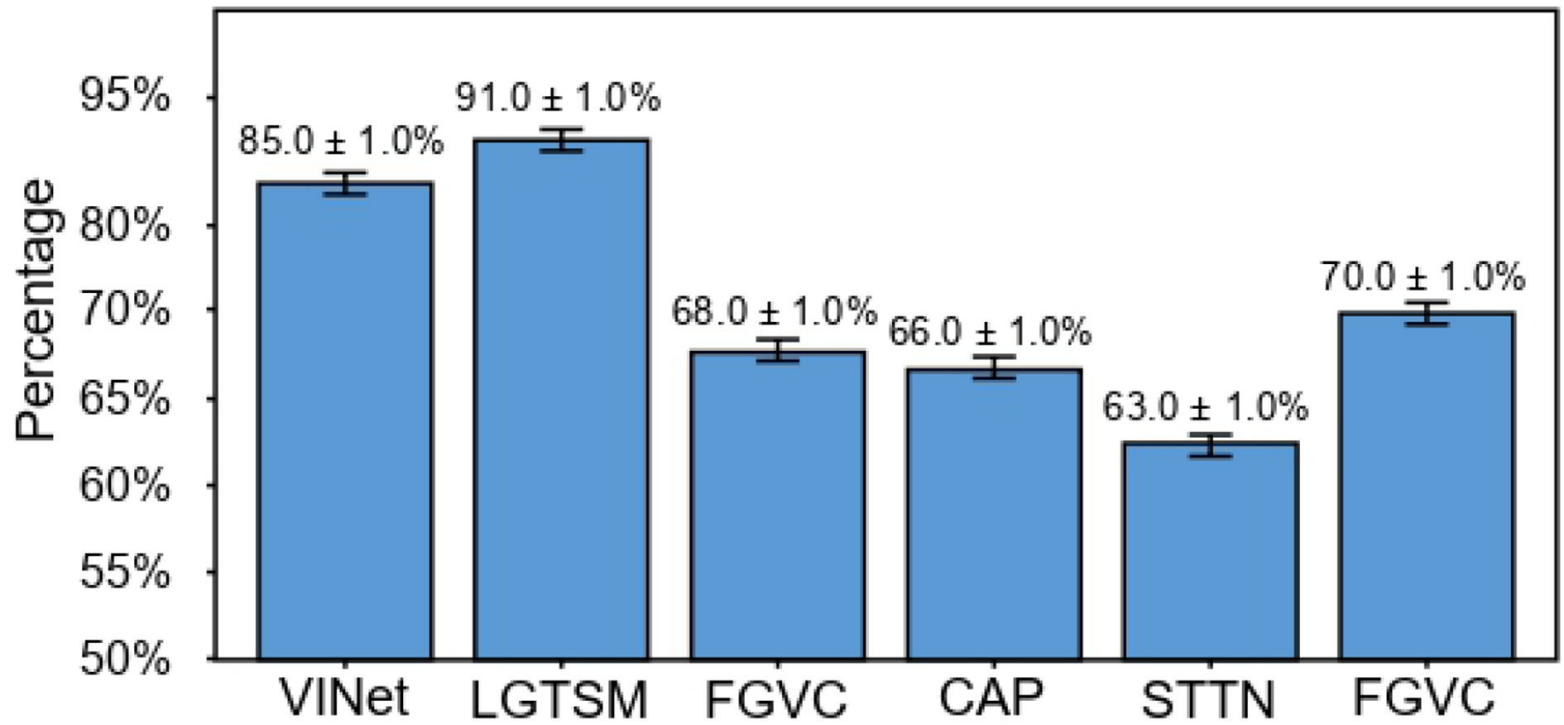
User study results for dynamic masks.

To eliminate the tendency of individuals to subjectively use a specific model, we selected 100 students in the school to conduct a user survey and gave each student 12 photos (a total of 7 comparison models, and each model selected two video restoration examples for the testing set). The students chose one image from the two repaired images containing STTTN each time they thought the repair was better and better; that is, each person made ten choices, for a total of 100 × 12 = 1, 200 voting choices. In Figure 5, the vertical axis represents the percentage for which they believe STTTN repair is better than the current model. The table shows that STTTN consistently outperforms other models compared with other inpainting effects.

### 4.3. Ablation studies

To verify the effectiveness of each part of STTTN, we carried out ablation learning, which is each part of texture transformation, and loss function, where each part of texture transformation contains four sets of ablation learning and the loss function contains two sets of ablation learning.

#### 4.3.1. Effects of various parts of texture transfer

As shown in Table 2, the texture transfer part is divided into four parts for ablation learning: CLFT, RHFT, encoder, and video frame prerepair. Base means removing these four parts and using the transformer for video repair (similar to a simplified version of STTN). We gradually increase these four parts each time to see the performance of STTTN. When CLFT and RHFT are added to the base, the PSNR increases by 0.25 and 0.19, respectively, so that STTTN can accurately convert the coarse low-frequency feature and refine the high-frequency feature and replace the finely designed encoder with ordinary Q, K, and V extraction. The improvement in PSNR is the most obvious (0.33), indicating that this part enables the texture converter to have more vital information extraction ability and fine content reconstruction ability. To explore whether it is helpful to pre-inpaint the reference frame and the input frame, we put them in the same domain, and the PSNR has a slight improvement of 0.03. In addition to the ablation learning of the four parts that make up the STTTN, we also added experiments to the experiment with regional normalization (RN) in the encoder and decoder to judge the impact of model performance. After adding RN, PSNR and SSIM were improved by 0.87 and 0.012, demonstrating the effectiveness of RN. The above ablation learning demonstrates the importance and effectiveness of the four parts, which complement each other and constitute a powerful texture transformer.

**Table 2:** Effects of various parts of texture transfer.

**Method**	**CLFT**	**RHFT**	**Encoder**	**Δ**	**RN**	**PSNR↑/SSIM↑/VFID↓/E_*w*_*arp↓***
Base					✓	30.52 / 0.9531 / 0.168 / 0.1810
Base+CLFT	✓				✓	30.77 / 0.9561 / 0.163 / 0.1793
Base+CLFT+RHFT	✓	✓			✓	30.96 / 0.9580 / 0.158 / 0.1774
Base+CLFT+RHFT+Encoder	✓	✓	✓		✓	31.29 / 0.9614 / 0.150 / 0.1741
Base+CLFT+RHFT+Encoder+Δ	✓	✓	✓	✓	✓	31.32 / 0.9620 / 0.149 / 0.1738
Base+CLFT+RHFT+Encoder+Δ	✓	✓	✓	✓		30.45 / 0.9500 / 0.151 / 0.1743

#### 4.3.2. Effects of charbonnier loss and transferal perceptual loss

**Figure 6:**
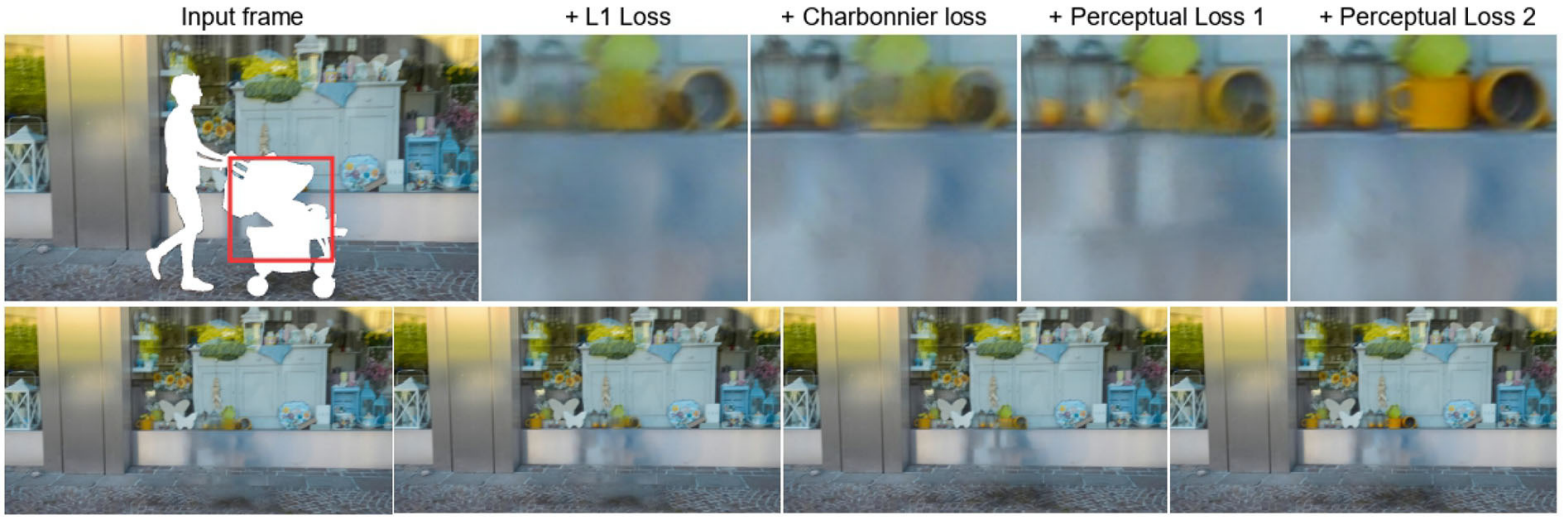
Effects of inpainting under different loss functions.

The five columns in the first row of Figure 6 represent the input frame with mask, use only the L1 loss function, replace the L1 loss with Charbonnier loss (C loss), add the first part of Perceptual Loss (P loss) based on the third column 1) based on column 4, and add the repair effect diagram of Part 2 (P loss 2) of Perceptual Loss. The second row is the uncropped inpainted frames for the four cases. Combining Figure 6 and Table 3, we can see that as we gradually complete the loss function, the effect of video repair gradually improves, which proves the effectiveness of each part of the loss function.

**Table 3:** Scores of STTTN under different loss function combinations.

**Method**	**L1 loss**	**Charbonnier loss**	**Perceptual loss 1**	**Perceptual loss 2**	**PSNR↑/SSIM↑**
L1 loss	✓				29.52 / 0.9395
C loss		✓			30.41 / 0.9532
C loss+P loss 1		✓	✓		31.12 / 0.9598
C loss+P loss 1+P loss 2		✓	✓	✓	31.29 / 0.9614

## 5. Conclusion

In this paper, we proposed a novel joint spatial-temporal texture transformer network for video inpainting. Each component cooperates closely, and the repair process progresses layer by layer, making full use of the texture feature information in time and space. The model has outstanding information extraction and content reconstruction capabilities in details and contours, which are essential and suitable for video repair tasks. The excellent results of STTTN's experiments on multiple datasets in multiple scenarios fully demonstrate its superiority over other methods.

However, in our exploration process, we found that STTTN has certain defects and room for further improvement. First, the first defect is also the region normalization defect, that is, color casting is prone to occur. The second defect, which is also a defect that the entire implicit inpainting architecture is prone to have, is the inconsistency in the temporal domain (the before and after frames have abnormal jitter effects during playback due to large local pixel changes, and blur artifacts appear in the video). Video tests appear periodically. We hope that our exploration of video inpainting can help other researchers explore further in this field and that researchers can propose more advanced models to improve the defects we have found thus far.

## Data availability statement

The original contributions presented in the study are included in the article/supplementary material, further inquiries can be directed to the corresponding author.

## Ethics statement

Written informed consent was obtained from the individual(s) for the publication of any potentially identifiable images or data included in this article.

## Author contributions

PM provided the idea of the algorithm and designed the entire architecture and was responsible for the writing of the manuscript and the conduct of the experiments. TX reviewed the manuscript. Both authors read and approved the final manuscript.

## Funding

This research was supported by the Shaanxi Provincial Technical Innovation Guidance Special (Fund) Plan in 2020 (2020CGXNG-012).

## Conflict of interest

The authors declare that the research was conducted in the absence of any commercial or financial relationships that could be construed as a potential conflict of interest.

## Publisher's note

All claims expressed in this article are solely those of the authors and do not necessarily represent those of their affiliated organizations, or those of the publisher, the editors and the reviewers. Any product that may be evaluated in this article, or claim that may be made by its manufacturer, is not guaranteed or endorsed by the publisher.
